# Complete Pathological Response After Neoadjuvant Chemo-Immunotherapy in Malignant Pleural Mesothelioma

**DOI:** 10.3389/fonc.2022.836751

**Published:** 2022-04-28

**Authors:** Francinne T. Tostes, Miguel Zugman, Vitor R. Paes, Gustavo Schvartsman

**Affiliations:** Centro de Oncologia e Hematologia Einstein Familia Dayan-Daycoval, Hospital Israelita Albert Einstein, São Paulo, Brazil

**Keywords:** mesothelioma malignant, chemo-immunotherapy combinations, checkpoint inhibition therapy, complete pathologic response (pCR), pleural mesothelioma, PD-L1 (22C3)

## Introduction

Malignant pleural mesothelioma (MPM) is a rare, aggressive disease that harbors a poor prognosis. Most patients are diagnosed with advanced disease, in which platinum-based combinations, with or without bevacizumab, yielded poor long-term outcomes. Recently, immune checkpoint inhibitors (ICI) have demonstrated promising activity for the treatment of MPM and have been incorporated as treatment options (1).

Around 20% of patients are eligible for attempted curative surgery at diagnosis. Treatment for such cases often involves multimodal therapy, including preoperative chemotherapy, followed by extrapleural pneumonectomy/pleural decortication and radiotherapy (2). Despite a comprehensive treatment, systematic reviews have demonstrated a median overall survival (OS) of only 13 to 23.9 months, likely due to the poor efficacy of systemic therapy, achieving 1.3% complete radiological responses and 5% pathological complete responses documented after neoadjuvant chemotherapy, warranting further improvement in the early-stage setting (3).

The combination of chemotherapy and immunotherapy showed promising results in several tumor types, with good tolerability. In advanced MPM, the combination of durvalumab with chemotherapy resulted in an objective tumor response rate of 48%, with a median overall survival of 18.4 months, higher than historical control with chemotherapy alone, irrespective of program death-ligand 1 (PD-L1) expression (4). Herein, we report a case of a borderline-resectable epithelioid pleural mesothelioma who underwent neoadjuvant therapy with cisplatin, pemetrexed and off-label pembrolizumab who was able to be operated, and obtained a complete pathological response (pCR) with sustained benefit, currently disease and treatment-free 14 months after surgery.

## Case Report

**Figure 1:**
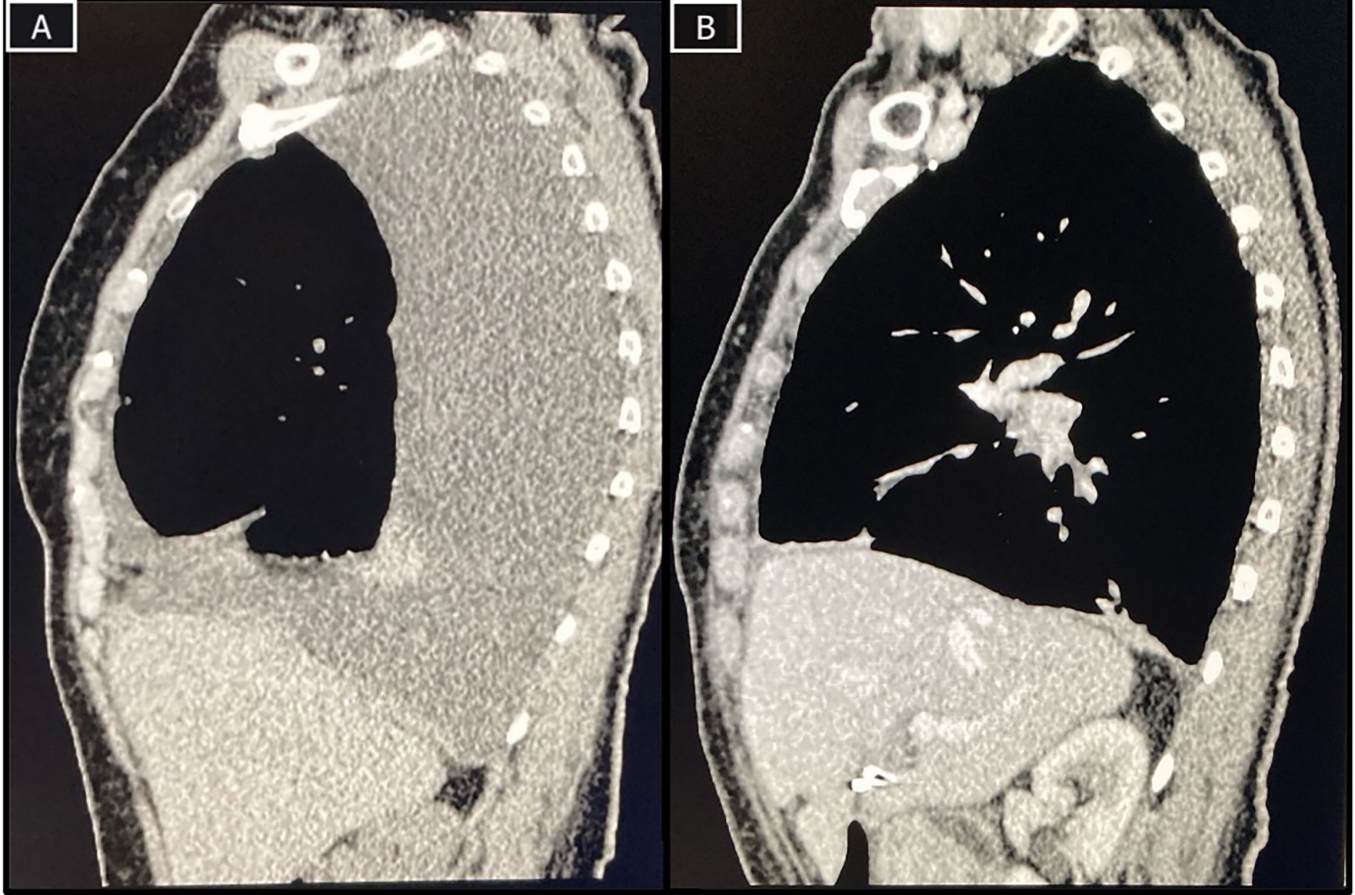
CT-scans at baseline and after surgery. **(A)** Baseline CT-scan demonstrating pleural effusion, thickening and nodules and a rounded atelectasis; **(B)** Restaging CT-scan with improvement of pleural effusion and minimal focal pleural thickening.

**Figure 2:**
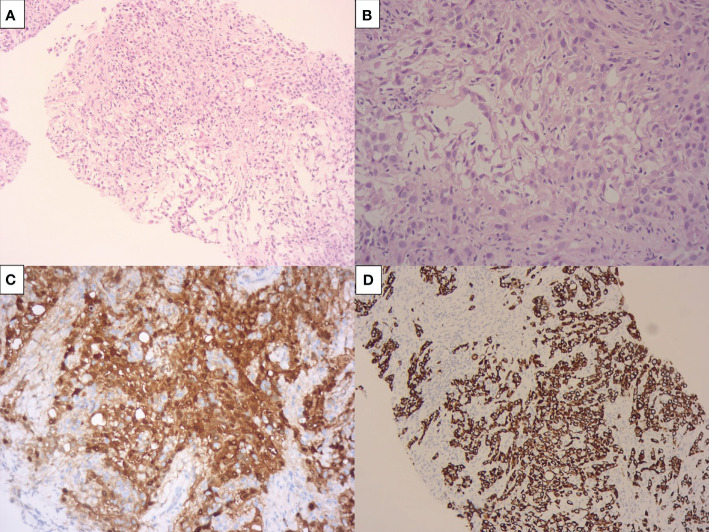
Pre-treatment pleural biopsy compatible with epithelioid malignant pleural mesothelioma. The pictures show a pleomorphic, solid epithelioid neoplasia **(A)**, with pseudoglandular formations and scattered lymphocytes **(B)**. This neoplasia was positive por CK7 **(C)** and calretinin **(D)** and negative por MOC31/BerEP4.

This is a 73-year-old female patient, non-smoker, with a prior history of occupational exposure to perchloroethylene during a 5-year period working at a dry-cleaning facility twenty years ago. She presented to the emergency department with a 4-week history of worsening dyspnea. She underwent pulmonary computed tomography (CT) that demonstrated a right-sided moderate pleural effusion, several nodules on the parietal, mediastinal and diaphragmatic ipsilateral pleura (**Figure 1A**). CT-guided biopsy of the pleura was compatible with epithelioid MPM (**Figure 2**).

**Figure 3:**
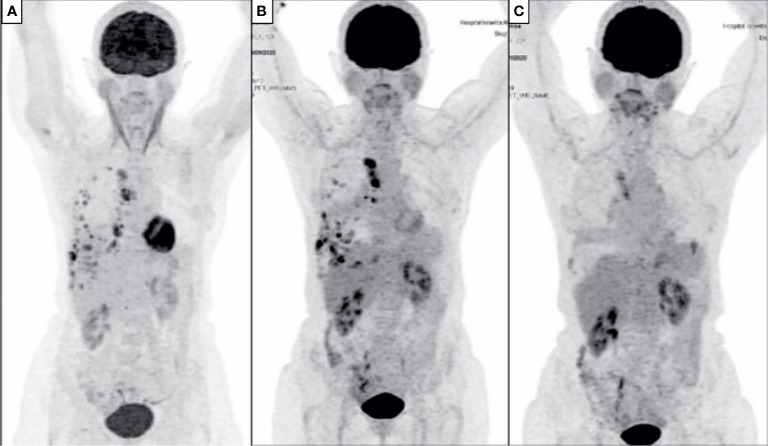
PET-CT at baseline and during treatment. **(A)** Baseline PET-CT demonstrating right pleural implants and mediastinal lymph node involvement; **(B)** restaging PET-CT after 2 cycles of treatment showing improvement in pleural nodules, but enlargement of lymph nodes; **(C)** PET-CT after 4 cycles showing complete improvement in pleural implants, and near-complete improvement of lymph nodes.

Positron emission tomography – computed tomography (PET-CT) and brain magnetic resonance imaging (MRI) were unremarkable for metastatic disease. The PET-CT did show, however, ipsilateral paratracheal and anterior mediastinal lymph nodes (**Figure 3A**). Clinical staging, according to the American Joint Committee on Cancer 8^th^ edition, was defined as cT1cN2M0. The mediastinal lymph nodes were not biopsied prior to therapy initiation.

**Figure 4:**
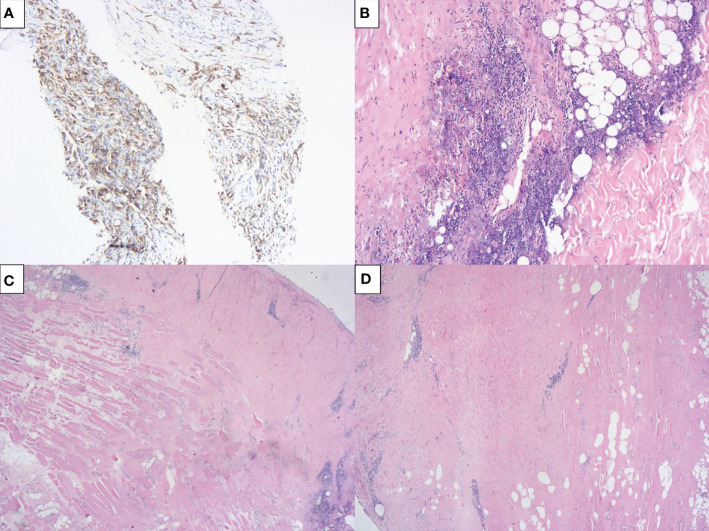
PD-L1 immunohistochemistry and pleural decortication slides. **(A)** Membrane PD-L1 expression on 80% of tumor cells (22C3 antibody) without significant lymphocytic infiltrate in the pre-treatment biopsy; **(B–D)** Pleural decortication slides showing a complete pathological response, characterized by fibrosis, multifocal chronic lymphocytic infiltrate and focal necrosis; no viable neoplastic cells were seen after exhaustive sampling.

The patient was considered borderline resectable due to local extent of disease, high symptom burden and patient frailty. A PD-L1 immunohistochemistry with 22C3 antibody clone was performed, resulting in an expression in tumor cells of 80% (**Figure 4A**). After discussion of risks and benefits, she was treated with cisplatin (75 mg/m^2^), pemetrexed (500 mg/m^2^) and pembrolizumab (2 mg/kg) every three weeks for 4 cycles, with no significant adverse events, except for tinnitus. An initial restaging PET-CT after 2 cycles showed a mixed response, with improvement in pleural implants but increase in the mediastinal lymph nodes’ FDG uptake and size (**Figure 3B**). Due to the patient’s clinical improvement, therapy was continued and a repeat scan after the fourth cycle demonstrated a complete metabolic response in pleural implants and near-complete improvement in the lymph nodes (**Figures 1B**, **3C**). Her case was discussed at multidisciplinary tumor board and she was considered eligible for surgical resection, no additional pathological exam was performed at the time prior to the surgical procedure. A pleural decortication and mediastinal lymph node dissection was performed and after thorough pathological review of the 105 slides, pCR was achieved in all sites (ypT0ypN0, **Figures 4B–D**). Adjuvant radiotherapy was omitted due to the pathologic response, and the patient is currently on surveillance with no evidence of disease after 14 months of her surgery and 18 months from systemic therapy initiation.

## Discussion

Treatment of advanced MPM had not seen major improvement in the last 20 years since the combination of cisplatin and pemetrexed was established as standard of care in the first-line setting, with a median overall survival of less than 12 months and less than 5% of patients remaining alive after 5 years (2). ICI have been studied in phase I/II trials in the refractory setting with promising response rates, though a randomized phase 3 trial published after our patient’s treatment decision failed to demonstrate improvement in progression-free and overall survival (5). Recently, CheckMate-743 evaluated the use of nivolumab plus ipilimumab in untreated patients, leading to a 26% reduction in the risk of death versus chemotherapy. At 2 years, 41% of patients were alive, compared with 27% who received chemotherapy (1).

PD-1 inhibitors are currently being investigated in several tumors as a neoadjuvant treatment, either as single agent, or combined with anti-CTLA-4 or chemotherapy. Pre-clinical and clinical data have postulated that early use of ICI may lead to improved efficacy, possibly due to a less immunosuppressive environment and enhanced priming of T-cells with the primary tumor’s antigens still present (6). In melanoma, the use of combination immunotherapy provided a 61% rate of pCR/near-pCR, with 96% of them relapse-free after 2 years (7). In non-small cell lung cancer, the combination of chemotherapy and nivolumab increased the pCR rate from 2% to 24%, compared with chemotherapy alone (8).

Thus, due to the overall poor prognosis of the disease, off-label strategies were discussed with the patient. Chemotherapy was warranted due to the potential resectability and high symptom burden. The rationale for the combination of chemotherapy and immunotherapy was based on the phase 2 DREAM trial, which treated patients in the advanced setting with durvalumab and standard chemotherapy, achieving a 48% overall response rate **(** 4 **).** Pembrolizumab was chosen due to reduced costs on a 2 mg/kg regimen, with comparable efficacy in other settings to a flat 200 mg dose. Our patient had an initial mixed response, with increased lymph nodes’ size and FDG uptake, a finding commonly seen with ICI, but less often seen when chemotherapy is associated. Pseudoprogression was reported in two cases in the DREAM trial. In such cases, this may represent a proof of concept that the immunotherapeutic agent likely played an important role in the documented efficacy. PD-L1 expression generally correlates with response to PD-1 inhibitors in several tumor types. Despite no clear association seen in mesothelioma trials, our patient had a high PD-L1 expression and an excellent response. After a pCR was achieved, no further treatment was administered and the patient is disease-free and well, corroborating the hypothesis of a durable benefit attributable to the anti-PD-1.

To the best of our knowledge, this is the first case report that illustrates the use of ICI in the neoadjuvant setting for MPM and the attainment of a pathological complete response. Further research in prospective clinical trials incorporating this strategy is warranted to improve clinical outcomes in patients with resectable tumors.

## Data Availability Statement

The original contributions presented in the study are included in the article/supplementary material. Further inquiries can be directed to the corresponding author.

## Author Contributions 

All authors listed have made a substantial, direct, and intellectual contribution to the work, and approved it for publication.

## Conflict of Interest

GS reports consultation fees from AstraZeneca, Merck, Sharp & Dome, Bristol-Myers Squibb, Sanofi Genzime, Novartis, Amgen and Roche.

The remaining authors declare that the research was conducted in the absence of any commercial or financial relationships that could be construed as a potential conflict of interest.

## Publisher’s Note

All claims expressed in this article are solely those of the authors and do not necessarily represent those of their affiliated organizations, or those of the publisher, the editors and the reviewers. Any product that may be evaluated in this article, or claim that may be made by its manufacturer, is not guaranteed or endorsed by the publisher.

## References

[1] Baas P Scherpereel A Nowak AK Fujimoto N Peters S Tsao AS. First-Line Nivolumab Plus Ipilimumab in Unresectable Malignant Pleural Mesothelioma (CheckMate 743): A Multicentre, Randomised, Open-Label, Phase 3 Trial. Lancet (2021) 397(10272):375–86. doi: 10.1016/S0140-6736(20)32714-8 33485464

[2] Cao CQ Yan TD Bannon PG McCaughan BC. A Systematic Review of Extrapleural Pneumonectomy for Malignant Pleural Mesothelioma. J Thorac Oncol (2010) 5(10):1692–703. doi: 10.1097/JTO.0b013e3181ed0489 20802345

[3] Krug LM Pass HI Rusch VW Kindler HL Sugarbaker DJ Rosenzweig KE. Multicenter Phase II Trial of Neoadjuvant Pemetrexed Plus Cisplatin Followed by Extrapleural Pneumonectomy and Radiation for Malignant Pleural Mesothelioma. J Clin Oncol (2009) 27(18):3007–13. doi: 10.1200/JCO.2008.20.3943 PMC3646305 19364962

[4] Nowak AK Lesterhuis WJ Kok PS Brown C Hughes BG Karikios DJ. Durvalumab With First-Line Chemotherapy in Previously Untreated Malignant Pleural Mesothelioma (DREAM): A Multicentre, Single-Arm, Phase 2 Trial With a Safety Run-in. Lancet Oncol (2020) 21(9):1213–23. doi: 10.1016/S1470-2045(20)30462-9 32888453

[5] Popat S Curioni-Fontecedro A Dafni U Shah R O’Brien M Pope A. A Multicentre Randomised Phase III Trial Comparing Pembrolizumab Versus Single-Agent Chemotherapy for Advanced Pre-Treated Malignant Pleural Mesothelioma: The European Thoracic Oncology Platform (ETOP 9-15) PROMISE-Meso Trial. Ann Oncol (2020) 31(12):1734–45. doi: 10.1016/j.annonc.2020.09.009 32976938

[6] Liu J Blake SJ Yong MC Harjunpää H Ngiow SF Takeda K. Improved Efficacy of Neoadjuvant Compared to Adjuvant Immunotherapy to Eradicate Metastatic Disease. Cancer Discov (2016) 6(12):1382–99. doi: 10.1158/2159-8290.CD-16-0577 27663893

[7] Menzies AM Amaria RN Rozeman EA Huang AC Tetzlaff MT van de Wiel BA. Pathological Response and Survival With Neoadjuvant Therapy in Melanoma: A Pooled Analysis From the International Neoadjuvant Melanoma Consortium (INMC). Nat Med (2021) 27:301–9. doi: 10.1038/s41591-020-01188-3 33558722

[8] Spicer J Wang C Tanaka F Saylors GB Chen K-N Liberman M. Surgical Outcomes From the Phase 3 CheckMate 816 Trial: Nivolumab (NIVO) + Platinum-Doublet Chemotherapy (Chemo) vs Chemo Alone as Neoadjuvant Treatment for Patients With Resectable Non-Small Cell Lung Cancer (NSCLC). J Clin Oncol (2021) 39(15_suppl):8503. doi: 10.1200/JCO.2021.39.15_suppl.8503

